# The status of international collaborations in compilation of Iranian scientific articles on environmental health engineering

**DOI:** 10.1186/s12992-019-0460-3

**Published:** 2019-02-27

**Authors:** Aram Tirgar, Seyed Ali Sajjadi, Zahra Aghalari

**Affiliations:** 1 Social Determinants of Health Research Center, Health Research Institute, Babol University of Medical Sciences, Babol, Iran; 2 Environment Health Engineering Department & Social Determinants of Health Research Centre, Gonabad University of Medical Sciences, Gonabad, Iran; 3 Environmental Health Engineering, Faculty of Health, Gonabad University of Medical Sciences, Gonabad, Iran

**Keywords:** Articles, Citation analysis, Environmental health, Journals, Research, Scientific participation, Scientometrics

## Abstract

**Background:**

Scientific cooperation is one of the effective methods to access current knowledge and technologies and also to use successful experiences of researchers in developed countries by academicians living in developing countries. This study aimed to determine the level of international contribution in compilation of scientific articles in Iranian journals published in the field of environmental health engineering.

**Methods:**

This descriptive and retrospective study was conducted within a 10-year period (2008–2017), in which all articles published in five specialized Iranian journals of environmental health engineering were reviewed using a researcher-made checklist. The information collected in the checklist included: the year of publication, number of issues and articles, information about the status of authors’ participation in terms of number of authors, sex, institutional affiliation, country, continents, and research centers. Descriptive and inferential statistics such as index of dispersion, measures of central tendency, and Chi-square and *t* tests were used to statistically analyze the data. Besides, VOSviewer software was used to visualize the data.

**Results:**

The review of 1276 articles published in 102 issues of the five journals of environmental health engineering in Iran showed that 184 articles were written with the participation of researchers from other countries. Most articles with the participation of international authors during the last decade were published in 2014. Chi-square test indicated a significant difference in the publication of these articles within 2014–2015 than other years (*P* = 0.001). Among the five journals, the best participation of international researchers was observed in J Environ Health Sci Engineer (168 articles, 91.3%). Considering the number of joint articles with Iran, the top continents were Asia, Europe, and Africa each with 117, 52, and 32 articles, respectively. India, Turkey, and Malaysia had the highest level of cooperation with Iranian researchers with 53, 16, and 14 articles, respectively. 637 authors contributed in 184 articles, of whom 469 (73.6%) were male and 121 (18.9%) were female. T test was used to compare the mean number of male and female authors in the articles with or without the participation of international researchers, which showed no significant difference.

**Conclusion:**

International contribution of researchers in compilation of specialized environmental health articles was good. Given the low level of cooperation between researchers from developed European and American countries and their Iranian counterparts, it seems necessary to adopt different methods to attract more collaboration from researchers working in developed countries considering their significant role in health-related areas.

## Background

The area of environmental health is one of the most prominent branches of medical sciences, which is classified as applied sciences and is taught in reputable universities worldwide [1]. This field has more than four decades of academic background in Iran with the history of about half a century as a profession. In the current world, 23% of deaths are related to environmental pollution [2]. As environmental health includes various sciences to prevent diseases, promote health, and provide human well-being, it can solve part of these environmental problems. The extended scope of environmental health activities in the terms of water, air, sewage, food, waste, and other interdisciplinary subjects necessitates students, researchers, and professors to cooperate with researchers working in other sciences and other countries by conducting appropriate studies and in accordance with the needs of the community to improve the quality of health services in various fields [3, 4]. The necessity of this issue has led to the fact that currently reputable journals of the field prefer to publish articles resulted from the collaborations of researchers from different countries [5]. So it is expected that studies and articles in the field of environmental health be conducted with the participation of a wider range of researchers from various national and international centers.

In recent years, various patterns of scientific cooperations (such as international and inter-institutional cooperations) have been growing rapidly among scholars and researchers [6]. Evidences show that the level of joint research has been increased among the experts in developing and developed countries, and the number of these scientific studies is still increasing [7]. Therefore, scientific cooperation can be considered as one of the effective ways to access the knowledge and technologies of developed countries in developing or newly developed countries. Besides, the increasing complexity of precise scientific tools, and the need to combine different types of knowledge and expertise to solve complex problems are also other essential motivations to perform such joint research. In addition, sharing knowledge and information, access to equipments and resources, division of labor, cost sharing, and increasing the quality of research are the other reasons for international cooperation [8, 9].

Since Iran is a developing country, part of its scientific advances in medical and health sciences are undoubtedly due to the opportunities provided by inter-institutional, academic, regional, and international cooperations [10]. In fact, considerable attention has been paid to science production with the participation of researchers from different national, regional, and international levels over the past years in Iran [10], and one of the criteria to evaluate the scientific activities of universities and research centers is the degree of participation of non-Iranian scholars and research centers in domestic scientific activities.

Scientometrics is an effective method to assess the extent of scientific contribution at various regional and international levels, as well as the quality of such scientific productions [11]. In fact, one of the standard tools for measuring and evaluating scientific productions in different scientific fields is using the Scientometrics. Scientometrics tools help to evaluate the quantitative and qualitative studies in different scientific fields, as well as the communication between researchers from different countries and institutions [12].

Scientometrics is defined as the knowledge of quantitative study of scientific disciplines based on published works and scientific relations. These studies may include identifying influential individuals and institutions in different fields, identifying the emerging areas of research, studying the development of disciplines over time, or geographical and institutional distribution of scientific products [13]. For example, a study conducted by Royle and colleagues entitled as “the level of collaborative researches done by Chinese scholars in international journals” showed that about half of their scientific products were the results of international contribution [14]. Hayati and co-workers also studied the level of contribution of Iranian researchers with other countries in indexed publications in the Citation Center within 1998–2007, and they found that Iranians had scientific contribution with 115 countries worldwide [15].

Given the importance of monitoring the science production process in each country, including in the field of environmental health, the main question is that “what is the status of researchers’ contribution in conducting studies in this field? How many studies in this area are performed with the participation of researchers from other countries? And what countries are mostly involved in Iranian research?”

The present study aimed to determine the status of national and international contributions in environmental health studies according to the articles published in specialized journals of the field during the past decade.

## Methods

This descriptive and retrospective study was conducted on all articles published in the specialized environmental health journals during a period of 10 years (2008–2017). The data were collected by referring to the websites of each journal from the beginning of 2008 to the last issue published in 2017.

The inclusion criteria were scientific research or specialized journal, having the term of environmental health in the title of the journal, having at least four issues per year, and the publication of articles for at least three consecutive years. According to these criteria, three Persian journals of “Iranian Journal of Health and Environment”, “Journal of Environmental Health Engineering” and “Journal of Research in Environmental Health”, as well as two English journals of “Environmental Health Engineering and Management Journal” and “Journal of Environmental Health Science and Engineering” were selected.

In the present study, the partnership means authors’ collaboration with authors and scholars from other countries or the presence of at least one international author in the articles. After visiting the dedicated websites of the journals, all articles were downloaded and the full texts of the articles were reviewed. The data collection was done by a researcher-made checklist in accordance with previous studies and using scientometrics [16, 17]. The checklist included the information on the status of authors’ participation in terms of number, sex, institutional affiliation, country and university, research centers according to the authors’ affiliation, as well as journal information divided by the years of publication, and number of issues and articles.

After collecting data, the information of all the articles were encoded and entered into an Excel file. Then, it was analyzed using descriptive and inferential statistics such as index of dispersion, central tendency, Chi-square, and *t* tests, and presented in the form of charts and tables.

VOSviewer software was also used to visualize the data and show the level of collaborations of continents and authors from different countries with Iran. This software helped to visualize the collaborative network of authors from different countries in different ways. For example, it makes it possible to display data aggregate by different colors, clustering them or their dispersion at different geographic locations [18].

## Results

In the present study, 1276 articles from 102 issues of five journals specialized in environmental science (three Persian-language and two English-language journals) were reviewed. Among the total number of articles published over the past 10 years, 36 issues and 626 articles were in English, and 66 issues and 650 articles were in Persian. Most of the Persian articles were related to the Journal of Health and Environment (430 articles, 66.1%) and the largest number of English articles were related to the J Environ Health Sci Engineer (JEHSE) (522 articles, 83.3%). Of all the reviewed articles, 184 papers (14.4%) were written with the participation of researchers from other countries.

**Fig. 1:**
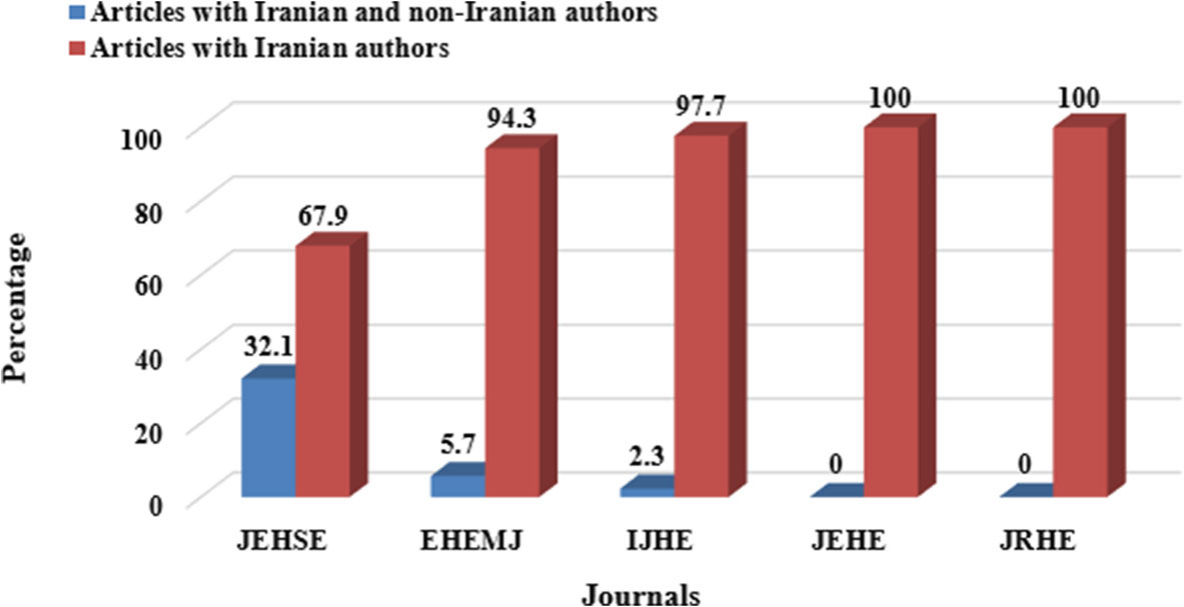
Relative frequency distributions of articles published in Iranian environmental health journals based on authors’ affiliations (2008–2017)

Comparing the proportion of articles published by international contribution in each of the reviewed journals showed that three journals published articles with international collaborations, and the largest number was observed in the JEHSE journal (168 articles, 91.3%). Figure 1 shows further information in this regard.

**Fig. 2:**
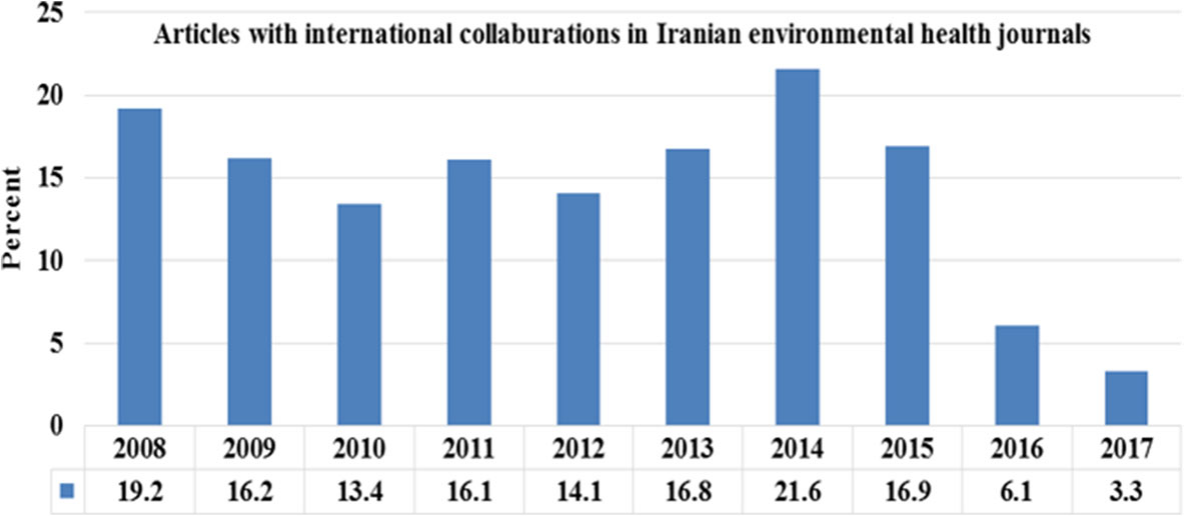
Relative frequency distributions of articles with international collaborations by Iranian environmental health journals based on publication’s year (2008–2017)

Figure 2 shows the process of publishing articles with international collaborations. As can be seen, during the past 10 years most articles of this type were published in 2014. Chi-square test showed a significant difference in the publication of articles of this type in 2014–2015 compared with other years (*P* = 0.001).

**Fig. 3:**
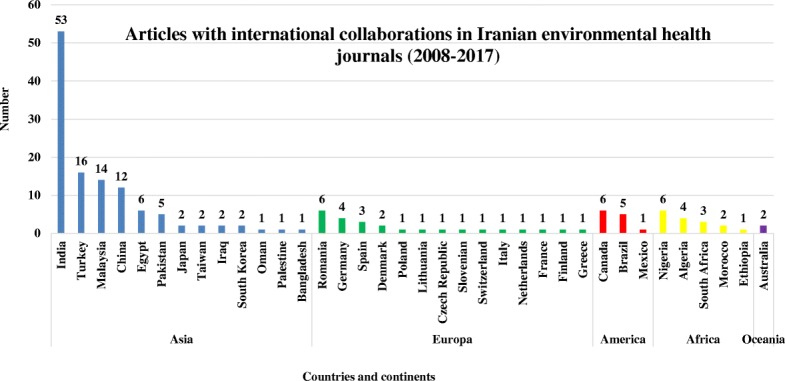
Frequency distributions of environmental health articles with the participation of Iranian and non-Iranian authors based on continents (2008–2017)

**Fig. 4:**
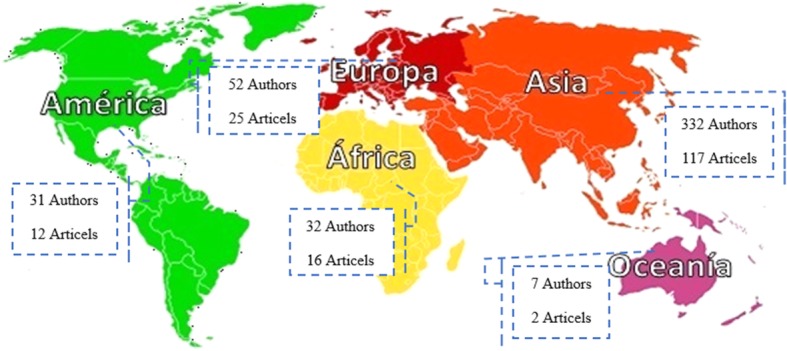
Global dispersion of authors of joint studies in the field of environmental health based on continents and countries (2008–2017)

Classification of 637 researchers participating in 184 joint papers according to the geographical location of the authors in five continents showed that 332 researchers with 117 articles (63.5%) were from 13 countries of the Asia. The classification of foreign authors of articles in terms of countries also showed that the largest number of researchers and articles conducting joint research with a frequency of 149 researchers and 53 articles was related to universities in India. It should be noted that among the reviewed articles, there were two papers from Australia (Oceania continent). Also, 12 articles were written by researchers from different continents. A total number of 505 international authors contributed in writing 184 articles (Figs. 3 and 4).

**Fig. 5:**
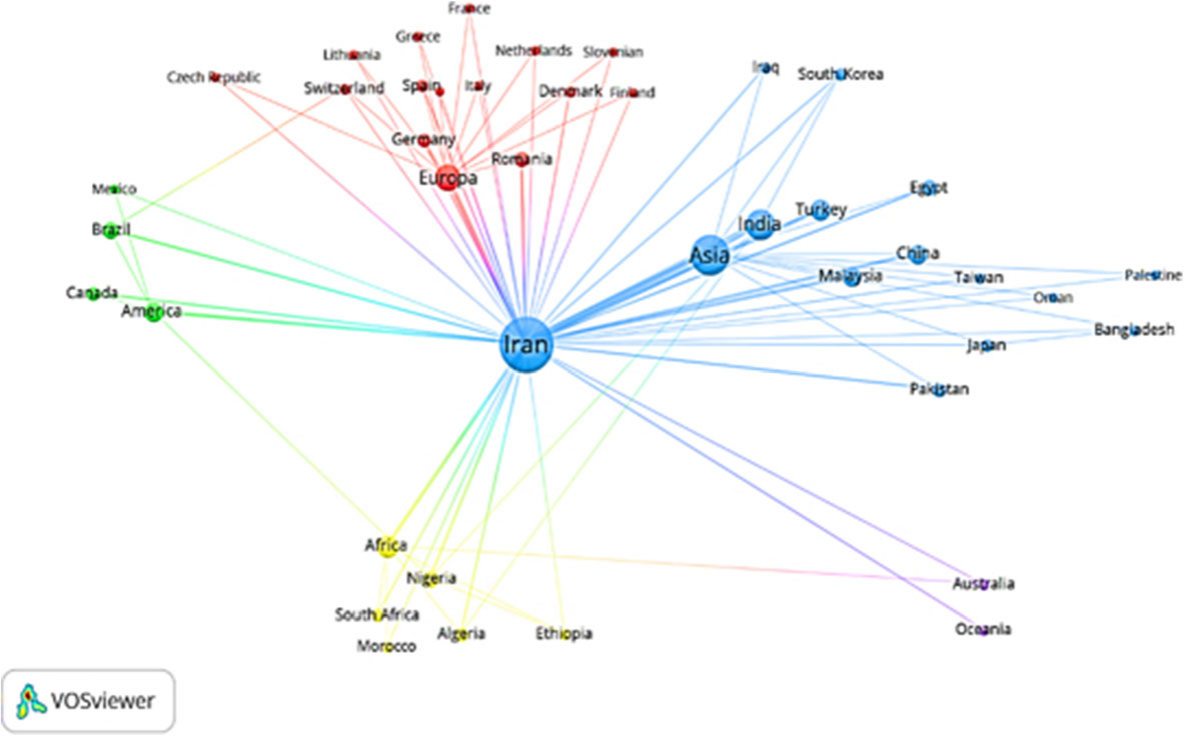
Collaborative network of authors of joint articles on environmental health based on continents and countries (2008–2017)

The relationships between authors of different countries and continents and Iranian authors in the compilation of articles in specialized environmental health journals and their collaborative network were visualized using 36 of VOS viewer software (Fig. 5). The findings suggested that there were at least 43 nodes (joint cooperations) in the compilation of articles, and 86 authors have collaborated with different geographic locations.

In this network, the size of each node represents the total number of articles of each country and continent collaborating with Iran, and the diameter of the bond between the two nodes is also proportional to the number of joint collaborations. Since the cooperation of foreign countries with Iran was our interest in this study, Iran had 148 articles in the center of the nodes. In this figure, countries more involved with Iranian authors are closer to Iran, and those countries with less participation are displayed at a distance.

According to the findings, the top continents based on the number of articles shared with Iran were Asia, Europe, and Africa each with 117, 52, and 32 articles, respectively. From the perspective of countries, India, Turkey, Malaysia, and China had the highest level of cooperation with Iranian researchers with 53, 16, 14, and 12 articles, respectively (Fig. 5).

According to the findings, among 1276 articles, 1092 articles were exclusively written by domestic researchers by collaboration of 4624 participants, and the number of authors was varied from 1 to 18 (4.21 ± 1.69). In 184 articles written by the participation of international researchers, more than two thirds of the papers were compiled with the participation of more than three researchers (128 papers, 69.5%). The number of foreign collaborators was 1 to 8 authors with the average number of 3.46 ± 1.50. T test for comparison between the average number of articles with and without the participation of foreign researchers showed no significant relationship (*P* = 0.603).

The sex distribution of the authors in 184 joint articles showed that among the total number of 637 authors, 469 (73.6%) were male and 121 (25.7%) were female, while the sex distribution of articles with domestic authors was 3198 (69.1%) male and 1223 (26.4%) female. It should be noted that the sex of 47 authors was not evident because only the authors’ last name was mentioned in the articles, given the similarity of the names of many authors, it was not possible to identify them through the search on the Internet. The minimum and maximum number of male authors in joint articles ranged from 0 to 7 (2.54 ± 1.64). The minimum and maximum number of female authors in joint articles ranged from 0 to 4 (0.65 ± 0.76). The minimum and maximum number of male authors in articles without the participation of foreign authors ranged from 0 to 14 (3.08 ± 1.64), and the minimum and maximum number of female authors in these articles ranged from 0 to 6 (1.12 ± 0.98). T test showed no significant difference in the comparison of the mean number of male and female authors in the articles with and without the participation of foreign researchers.

Among the articles with joint international collaborations, environmental health engineering was the specialized field of the first author in 16 papers (8.7%) and in 17 articles (9.2%) was the specialized field of the corresponding author. The first or corresponding authors in other articles were specialists in other fields such as environments, occupational health, public health, epidemiology, etc. Table 1**.**

**Table 1:** Distribution of corresponding and first authors with environmental health engineering expertise in articles published in Iranian environmental health journals

Type of article	Frequency	Environmental health authors		
		Articles in which the corresponding or first author was environmental health specialist (%)	*First author (%)	*Corresponding author (%)
Persian articles with only domestic authors	650	397 (61)	369 (56.7)	356 (54.7)
English articles with only domestic authors	626	247 (39.4)	225 (36)	238 (38)
articles in Persian or English with foreign authors	148	18 (12.1)	16 (10.8)	17 (11.5)

*The inequality of the number and percentage of the corresponding and first authors is due to the fact that in some articles the first and corresponding authors were both specialized in environmental health

Among 184 articles with international collaborations, the affiliations of 78 individuals in 35 articles were listed as the member of a research center. Generally, 30 research centers were mentioned in the articles, 14 cases were Iranian centers. Among the research centers in Iran, the highest number belonged to Tehran University of Medical Sciences with five centers. There were also 16 foreign research centers and the highest number belonged to Indian universities. More information is provided in Table 2.

**Table 2:** Frequency of authors’ participation in terms of research centers in articles published in the Iranian environmental health journals

Country and City		Research center	Number
Iran	Tehran	Environmental Health Center	15
		Occupational Health Research Center	3
		Technology Incubation Centre	1
		Ecology Centre	1
		Pregnancy Health Centre	1
	Tabriz	Environmental Engineering Research Center	3
	Ahvaz	Environmental Health Center	3
	Kerman	Environmental Health Center	1
	Zahedan	Health Center	1
	Boshehr	Biotechnology Research Center	1
	Kordestan	Environmental Health Center	1
	Shahrood	Environmental Engineering Research Center	1
	Azad university of Tehran	Young Researchers Club	1
	University of Tehran	Environmental Biotechnology	1
Other countries	India	Centre for Environmental Studies	13
		Coordinator of International Centre for	4
		Ecological Engineering	2
		Centre for Energy Studies	1
		Desert Medicine Research Centre	1
	Romania	Modern Dental College & Research Center	5
		The Interdisciplinary Centre for Advanced Researches on Territorial Dynamics	2
		National Institute of Research and Development for Biological Sciences	1
	Switzerland	Research Institute for Analytical Instrumentation	4
	Egypt	Swiss Tropical and Public Health Institute	4
	Malaysia	Environmental	3
	America	Engineering Research Centre	2
	China	Environmental	2
	Japan	Engineering Research Centre	1
	Japan and Malaysia	Center for Environmental Nanoscience and Risk	1
	Australia	Geology Center	1

## Discussion

The findings of the status of scientific contributions in the articles of the five specialized journals of environmental health in Iran during 2008–2017 showed that among the 1276 articles published in this period, 184 articles had 14.4% participation of researchers from other countries and the best performance in this regard belonged to JEHSE with 168 articles (91.30% of this type of articles and 13.1% of the total articles). Maybe the main reason for publishing joint articles in this journal was that the journal was indexed in ISI and PUBMED, and domestic researchers with their foreign co-authors were more interested in publishing their articles in such an indexed journal. In a study by Zhang and colleagues in China, using a method of scientometrics emphasizing health management research (using “Health Management” keyword in the “Web of Knowledge” from 1999 to 2011), it was found that the status of collaboration between Chinese researchers and authors from other countries, was about 13%, which was relatively similar to the present study [19]. They stated that despite the growing international cooperation in compiling articles on health management, it was necessary to increase the level of cooperation between scientific institutions and universities at the national and international levels, especially on the top areas of research [19].

Royle and co-workers also reviewed the level of collaborative productions in more than 35,000 Chinese studies in journals published by the Elsevier in 2004 and concluded that about 20% of the studies were produced by international cooperation with scholars from other countries. Their findings showed that in 49% of joint articles with other countries, the corresponding author was Chinese. Besides, the geographical proximity had an effect on the increased cooperation [14], which showed the influential role of the geographic position of countries and their proximity on the level of scientific cooperation.

Hayati and colleagues also investigated the level of collaboration between Iranian researchers and other countries in journals indexed in the ISC database during 1998–2007, and revealed that Iranians collaborated with 115 countries. Their results showed that the number of articles written by the cooperation with domestic institutions was three times more than international cooperation. Most international cooperation was observed in geosciences, and most important partners were from the United States, Canada, and England, respectively [15].

Zyoud and others in a study on the comparison of the quality and quantity of publications by Arab researchers in the field of pharmaceutical waste in the Scopus database reported that international cooperation for scientific publications on sewage was more among Arab countries and European countries than Asian countries (30.2% versus 16.2%). Of course, this can be due to the role of scientific and educational contributions of European countries in Arab countries [20]. Generally, international cooperation in the articles of the field of environmental health compared with their Asian counterparts shows that Iran’s scientific status is less satisfactory than other countries in the world and the region, which emphasizes the need for serious attention to this issue. In fact, a group of Iranian researchers, including environmental health researchers, use international collaboration in their studies, but they publish their articles in more reputable foreign journals in order to increase the visibility of their articles and gain higher rates. According to this fact, if the level of scientific cooperation was examined in all international articles of environmental health, probably better results would obtain.

Soteriades and colleagues in an analysis of articles from 1995 to 2003 in ISI database on professional and general environmental health showed that American countries ranked first in the this area with 60% of published articles, and the European countries ranked the second [21]. So, regardless of the level of international participation, the choice of country and continent of scientific partners is very important. Therefore, it is better to develop partnerships with USA and developed European countries in order to extend international partnerships in scientific and research activities.

The findings of the present study showed that the contribution of Iranian authors with authors from other countries was reduced over the years 2016 and 2017. It seems that the main reason for this reduction is the political status and social relations between countries.

The classification of 184 article written by 637 researchers from 39 countries (including Iran) showed that the largest number of articles (117, 63.5%) was from the 13 Asian countries, which were written with the collaboration of 332 non-Iranian scholars. One of the reasons for high participation of Asian scholars in the publication of scientific articles in Iran may be that Asia is the most populous continent in the world with many environmental problems affecting human health for a long time [22]. Another reason can be the proximity of Asian countries to Iran. Besides, many Asian countries have a friendly political relationship and extended cooperation with Iran, which can be another reason for the high level of participation among Asian scholars in this study.

However, it should be noted that the limited participation of Iranian scholars with European and American countries can be due to political or even economic issues. As it was revealed in the study by Tabatabaei and colleagues on the publication of herbal medicines articles from 1997 to 2014, the process of publication of herbal medicines articles was growing, but it declined since 2014, after imposing sanctions against Iran [23]. The decline in the number of collaborations after 2014 is approximately similar to the present study (Fig. 1).

Among the total number of 184 articles reviewed, 61 articles (33.1%) were produced collaboratively by several universities and academic centers. It shows the good status of the participation of researchers from different scientific centers, and suggests proper attention paid to maintain and expand such collaborations in conducting research. The more the number of scientific centers in the implementation of a research project, the more the quality or speed of performing research is improved.

Pless and co-workers in a review on the need for research on chronic diseases in children, concluded that the collaboration of scientific centers in research would facilitate funding for research projects, as well as reinforce implementing more research [24]. The positive impact of such partnerships in Iran is also predictable due to the existence of various universities (Public, Azad, Non-profit, and Payam-Noor Universities) with independent managements and resources.

The study of the status of research participation in other sciences in publication of international articles in the area of environmental health showed that in 18 cases (12.1%), the first or corresponding authors was environmental health professional. This proportion was 638 (50%) in all articles (1276 articles). More participation of professionals of different scientific fields and research centers can improve the quality, funding, research opportunities, and production of new ideas and can reduce research errors.

Resnick, in a research entitled “Increasing Opportunities through Interdisciplinary Research”, found that if studies were carried out by cooperation of interdisciplinary researchers, in addition to integrating ideas, it could reduce the percentage of errors [25]. Therefore, it would be hoped that increased collaboration of environmental health professionals with other researchers in the domains such as occupational health, environment, basic medical sciences, and engineering could open up new opportunities and create innovations in environmental health. Given the positive effects of scientific contributions, developing logically and accountably cooperation with other sciences should be considered.

Based on the findings of this study, the mean number of authors in all articles was 4.10 ± 1.68, and this number was 3.46 ± 1.50 for the articles with the participation of foreign researchers, which shows the better level of collaboration between the authors of environmental health articles in the domestic journals than joint researches with other countries.

Sex distribution of articles showed that the ratio of male to female authors in internal articles was 69.1% versus 28.4%; while in the journals with the participation of foreign researchers this ratio was 73.6% versus 25.7%. This shows lower proportion of female researchers in all papers, especially in international papers. Leading countries in performing research have focused on sex balance among scholars, and even some research organizations in the United States and Switzerland have been planning to engage more women in research projects [26]. The study of Sidhu on the level of women’s participation in writing articles in six British medical journals, found that women’s participation rate increased by 4.2% over the 35 years [27]. Amrein’s study on the 60 journals of medical sciences in the Reuters database revealed that women’s presence in the editorial board of medical sciences journals was very low, so that there are only 15.9% female editors meaning less than one-fifth of the editorial staff among 60 journals [28].

Since the topic of international partnerships in different areas of medical sciences in Iran has received less attention, so examining the issue of national and international partnerships in environmental health can be the strength point of the present study. We need to try harder on developing scientific and research partnerships with developed countries to achieve scientific improvement, particularly in the health sector. The weaknesses of the present study is the lack of access to all articles in the environmental health domain, because some environmental health researchers publish their articles in foreign journals or other domestic medical journals that are not merely relevant to environmental health and naturally required more time and budget to search for them in those journals.

Considering the importance of expanding collaboration between researchers in Iran and other countries, especially developed countries, it is recommended to consider improving the quality of research in various ways, including:

- Making cooperation agreements between scientific centers,
- Deploying talented and capable scholars to leading institutions and centers,
- Gaining the trust of other researchers from other countries to perform genuine, extensive, and robust research plans, and
- Allocating adequate budget to research projects, especially incentive measures for studies with the participation of researchers from other countries.

## Conclusion

According to the available statistics in the region, during the specified period, the level of international contribution in the compilation of specialized environmental health articles was relatively good, but due to the lack of continuation of this trend over the last two years and the lack of cooperation of developed European countries in domestic projects, it seems necessary to adopt different methods in order to attract more collaborative researchers from developed countries considering their pioneering role in health-related areas.

## Abbreviations

EHEMJ: Environmental Health Engineering and Management Journal
IJHE: Iranian Journal of Health and Environment
ISC: Inter-Species Conserved
ISI: Institute for Scientific Information
JEHE: Journal of Environmental Health Engineering
JEHSE: Journal of Environmental Health Science and Engineering
JRHE: Journal of Research in Environmental Health
